# Tau excess impairs mitosis and kinesin-5 function, leading to aneuploidy and cell death

**DOI:** 10.1242/dmm.022558

**Published:** 2016-03-01

**Authors:** Anne-Laure Bougé, Marie-Laure Parmentier

**Affiliations:** Department of Neurosciences, Institut de Génomique Fonctionnelle, CNRS-UMR5203, INSERM-U1191, Université Montpellier, 141 Rue de la Cardonille, Montpellier F-34094, Cedex 5, France

**Keywords:** Alzheimer's disease, *Drosophila* genetics, Eg5 (KIF11) kinesin, MAPT protein, Neurodegenerative diseases, Aneuploidy

## Abstract

In neurodegenerative diseases such as Alzheimer's disease (AD), cell cycle defects and associated aneuploidy have been described. However, the importance of these defects in the physiopathology of AD and the underlying mechanistic processes are largely unknown, in particular with respect to the microtubule (MT)-binding protein Tau, which is found in excess in the brain and cerebrospinal fluid of affected individuals. Although it has long been known that Tau is phosphorylated during mitosis to generate a lower affinity for MTs, there is, to our knowledge, no indication that an excess of this protein could affect mitosis. Here, we studied the effect of an excess of human Tau (hTau) protein on cell mitosis *in vivo*. Using the *Drosophila* developing wing disc epithelium as a model, we show that an excess of hTau induces a mitotic arrest, with the presence of monopolar spindles. This mitotic defect leads to aneuploidy and apoptotic cell death. We studied the mechanism of action of hTau and found that the MT-binding domain of hTau is responsible for these defects. We also demonstrate that the effects of hTau occur via the inhibition of the function of the kinesin Klp61F, the *Drosophila* homologue of kinesin-5 (also called Eg5 or KIF11). We finally show that this deleterious effect of hTau is also found in other *Drosophila* cell types (neuroblasts) and tissues (the developing eye disc), as well as in human HeLa cells. By demonstrating that MT-bound Tau inhibits the Eg5 kinesin and cell mitosis, our work provides a new framework to consider the role of Tau in neurodegenerative diseases.

## Abstract (precis)

***Drosophila* Collection:** We show that Tau, a microtubule-binding protein involved in many neurodegenerative diseases, impairs mitosis when in excess. We show that this occurs via the inhibition of the kinesin-5 mitotic motor.

## INTRODUCTION

Alzheimer's disease (AD) is a complex, progressive and irreversible neurodegenerative disease of the brain, and the most common form of dementia in the elderly. Symptoms start when neurons in brain regions involved in memory, cognition and neurogenesis are being damaged and ultimately die. The hallmark pathological lesions of the disease are extracellular senile plaques (SPs) and intraneuronal neurofibrillary tangles (NFTs). Whereas the SPs are composed of beta amyloid peptide (Aβ), which is the product of abnormal processing of APP protein (amyloid precursor protein), the NFTs are composed of the microtubule (MT)-associated protein Tau (MAPT). Within the NFTs, the Tau protein is found hyperphosphorylated, with phosphorylation on many more residues than normally occurs (Grundke-Iqbal et al., 1986). More generally, neurodegenerative disorders with intracellular Tau filamentous deposits are referred to as tauopathies (Delacourte and Buée, 2000; Lee et al., 2001). These include, in addition to AD, progressive supranuclear palsy, corticobasal degeneration, Pick's disease and argyrophilic grain disease, as well as the inherited frontotemporal dementia and parkinsonism linked to chromosome 17 (FTDP-17). The identification of mutations in Tau as the cause of some of these tauopathies (e.g. FTDP-17 frontotemporal lobar degeneration with Tau inclusions) has further indicated the important role of this protein in neurodegeneration (Frost et al., 2015).

Two decades ago, chromosome missegregation was proposed to be responsible for neurodegeneration in individuals with AD. Indeed, such individuals develop up to 30% aneuploid or polyploid cells both in brain and peripheral tissues, indicating the presence of widespread chromosome partitioning defects (Iourov et al., 2009; Migliore et al., 1997; Mosch et al., 2007; Yurov et al., 2014). Furthermore, the aneuploid and hyperploid neurons that arise in AD are particularly prone to degeneration and could account for 90% of the neuronal loss that characterizes late-stage AD (Arendt et al., 2010). Several causes could explain the excess of aneuploidy in AD brain: (i) lack of aneuploidy clearance during brain development, (ii) an increased propensity for chromosome missegregation during mitosis during development and in the adult or (iii) an aberrant attempt of cell cycle re-entry. The fact that peripheral blood lymphocytes of individuals with AD are prone to undergo aneuploidy spontaneously (Migliore et al., 1997) is in favour of the second hypothesis, i.e. an increased general propensity for chromosome missegregation. Further evidence for the potential involvement of cell cycle defects in AD comes from the fact that both APP and Tau are increasingly phosphorylated during mitosis (Pope et al., 1994; Preuss et al., 1995; Suzuki et al., 1994). This suggests that the physiological regulation of the phosphorylation of these proteins is important for the correct progression of mitosis. In accordance with this idea, it was recently shown that an excess of Aβ can actually induce mitotic spindle defects and consequent aneuploidy (Borysov et al., 2011). Such a deleterious role of an excess of Tau on mitosis was never shown, although recent data show an increased level of aneuploidy in splenic lymphocytes of transgenic mouse models of tauopathies (Rossi et al., 2014). It was also reported that individuals with the TauP301L mutation, which is associated with frontotemporal dementia, had several chromosome aberrations, such as aneuploidies in their fibroblasts and lymphocytes (Rossi et al., 2008), raising the question of the cellular mechanisms involved.

Here, we studied the effect of an excess of human Tau (hTau) protein on cell mitosis *in vivo*. Using the *Drosophila* developing wing disc epithelium as a model, we show that an excess of hTau induces a mitotic arrest, with the presence of monopolar spindles. This mitotic defect leads to aneuploidy and apoptotic cell death. We studied the mechanism of action of hTau and found that the MT-binding domain of hTau is responsible for these defects. We also demonstrate that hTau effects occur via the inhibition of the function of the kinesin Klp61F, the *Drosophila* homologue of Eg5 (also known as KIF11). We finally show that this deleterious effect of hTau is also found in other cell types (neuroblasts) and tissues (the developing eye disc) as well as in cell culture. Altogether, our results show that an excess of hTau strongly impairs cell division and that this effect involves the hTau domain that binds to MT and the inhibition of Klp61F/Eg5 function.

## RESULTS

### hTau overexpression in epithelial cells induces mitotic defects

**Fig. 1.**
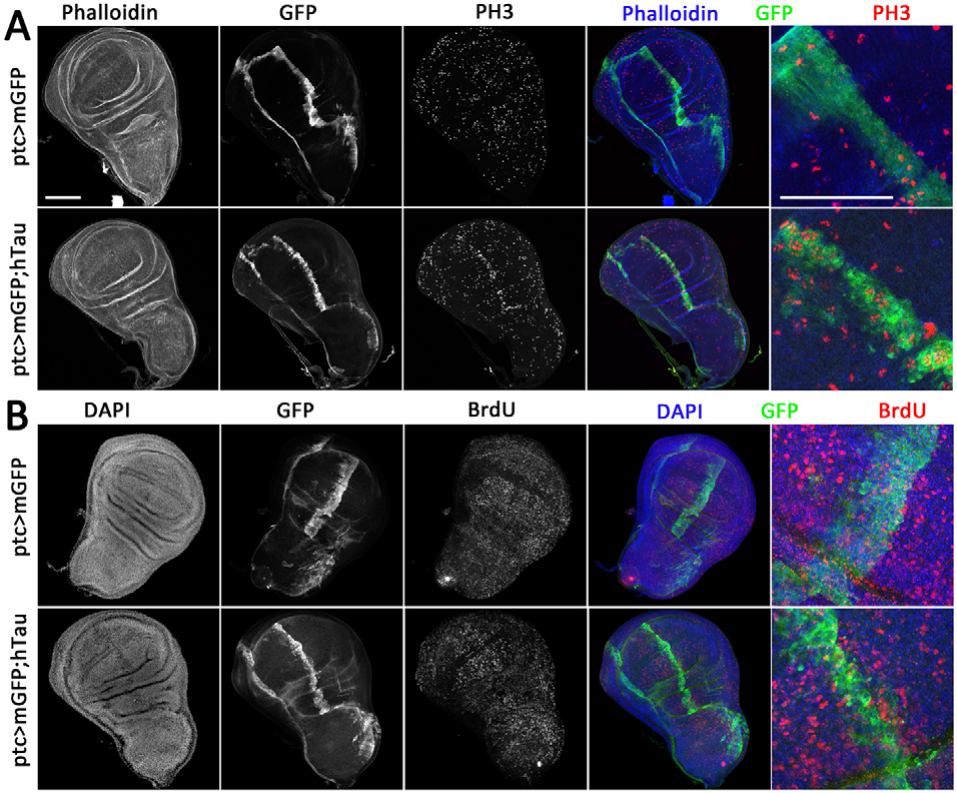
**hTau overexpression affects mitosis in the wing disc.** Immunostainings of third instar larval wing discs overexpressing human Tau  (hTau) protein 0N4R in the *ptc* domain of the disc. As a control, a membrane-targeted GFP (mGFP) is expressed alone in the same region. (A) GFP (green), PH3 (red) and phalloidin (blue) triple staining. There is a homogeneous repartition of cells undergoing mitosis (PH3-positive) within control wing discs overexpressing mGFP in the *ptc* domain (ptc>mGFP, top row). When hTau is overexpressed in addition to mGFP in the *ptc* domain (ptc>mGFP;hTau, bottom row), there is an increase of PH3 staining within the *ptc* domain, as is visible at low magnification (four left panels). High magnification of the *ptc* domain (right panel) shows an increase in the number of PH3 spots within this domain in the presence of hTau. (B) GFP (green), BrdU (red) and DAPI (blue) triple staining. There is a homogeneous repartition of cells having undergone S phase (BrdU-positive) within control wing discs overexpressing mGFP in the *ptc* domain (ptc>mGFP, top row). There is no change of this repartition within the *ptc* domain when hTau is overexpressed in addition to mGFP in this region (ptc>mGFP;hTau, bottom row). Scale bars: 50 µm. These experiments were replicated at least three times in the laboratory.

In order to study the effect of hTau on dividing cells, we focused on the *Drosophila* wing imaginal disc, which consists of one columnar epithelium. During the larval stages, many cell divisions take place in this epithelium as it grows in size to form the future adult wing. We overexpressed hTau, together with GFP, in a specific area of the wing disc (see the GFP staining in Fig. 1A,B and in Fig. S1), using the *ptc-Gal4* driver. The *hTau* transgene that we used in this work is the 0N4R Tau splice variant (Andreadis et al., 1992; Goedert et al., 1989; Kosik et al., 1989), which we tagged with a flag tag at the C-terminus (Fig. S1). We first tested whether an excess of hTau in the *ptc* expression domain affected the cell cycle by looking at the number of cells undergoing mitosis (PH3-positive cells) in this area. There was a clear increase in PH3 staining in the *ptc* area (Fig. 1A), as measured by 14±2.1% of PH3-positive pixels in this area, compared to 2.7±0.6% of PH3-positive pixels outside this area within the wing pouch (*n*=5; *P*<0.001). This observed excess of cells undergoing mitosis in the presence of hTau could be due either to a change in the cell cycle duration (a shorter cell cycle would result in more cells undergoing mitosis) or to defects in mitosis (mitosis would take longer). We tested the first hypothesis by performing BrdU staining, which labels cells undergoing S phase. We could not see any clear increase in the number of BrdU-positive cells in the *ptc* domain where hTau is expressed (Fig. 1B), indicating that there is no shortening of the cell cycle duration.

### Tau excess disrupts the mitotic spindle by inducing monopolar spindles

**Fig. 2.**
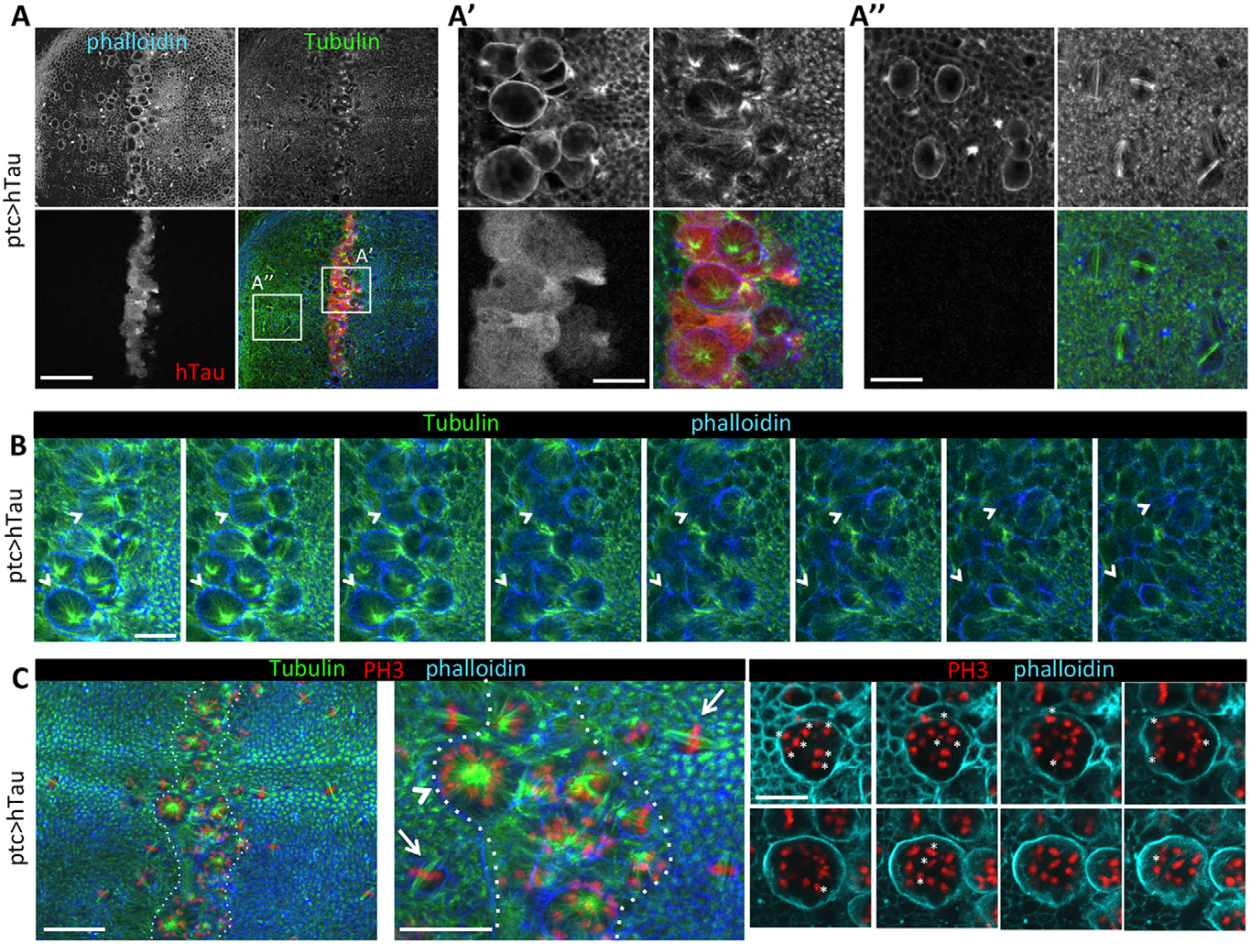
**hTau overexpression induces monopolar spindles and chromosomal aneuploidy.** (A,B) Immunostainings of mitotic spindles in third instar larval wing discs overexpressing human Tau (hTau) protein 0N4R in the *ptc* domain: Tubulin (green), hTau (red) and phalloidin (blue). (A) Cells within the *ptc* domain are large, like dividing cells outside the *ptc* area, as seen with the phalloidin staining at low magnification. A higher magnification of the *ptc* area (A′) shows that those large cells correspond to mitotic cells, with a spindle that seems mis-oriented compared to dividing cells present outside the *ptc* area (A″), because only one spindle pole is clearly visible in a confocal section. Scale bars: 30 µm in A, and 10 µm in A′,A″. (B) A *z*-series of confocal sections of the *ptc* area shows that the opposite spindle pole of hTau-overexpressing cells is never visible (see putative positions of opposite pole indicated by arrowheads for two selected cells). This indicates that these cells actually display abnormal monopolar spindles. Scale bar: 10 µm. (C) Immunostainings of mitotic spindles and mitotic chromosomes in third instar larval wing discs overexpressing hTau protein 0N4R in the *ptc* domain: Tubulin (green), PH3 (red) and phalloidin (blue). Left panel: the *ptc* area (delimited by dotted lines) contains many large cells with monopolar spindles and PH3-positive chromosomes. Middle panel: a higher magnification of the *ptc* area shows that those large cells with monopolar spindles contain a high number of chromosomes. Outside the *ptc* area are cells dividing normally (arrows), with a normal content of chromosomes. Right panels: a z-series of confocal sections of one cell within the *ptc* area (see arrowhead in the middle panel) allows the precise counting of chromosomes, each new chromosome on the next *z*-section being highlighted by a white star: the total number of chromosomes for the studied cell is 20, a number much larger that the eight chromosomes expected from a single DNA replication of the four *Drosophila* chromosomes. Scale bars: 20 µm in the left panel and 10 µm in the middle and right panels. These experiments were replicated at least three times in the laboratory.

Hence, there might be a defect in mitosis, which we further studied in detail by looking at the mitotic spindle. We stained mitotic spindles using anti-tubulin antibodies. In normal conditions, cell divisions within the wing disc occur with a planar alignment of the mitotic spindle such that there is symmetric cell cleavage (Fig. 2A-A″ and Fig. S2). Here, in presence of an excess of hTau in the *ptc* domain, we could not see planar spindles and, in particular, we could only see one pole very clearly (Fig. 2A′). In order to test whether this was due to a change in the spindle orientation, we searched for the other spindle pole in serial confocal sections (Fig. 2B). This other pole was not visible, indicating that these cells actually have monopolar spindles (Fig. 2B). We tested whether this defect could be seen when using different *Gal4* lines expressing in different areas of the wing disc. We could see this defect with *dpp-Gal4*, which expresses hTau in a broader area of the wing (Fig. S3). We could also see this defect when expressing hTau in the whole wing disc with *MS1096* as a *Gal4* driver (Fig. S3). This indicates that this defect can be observed when hTau is expressed at any place within the wing disc. In conclusion, an excess of human Tau induces a strong alteration of the mitotic spindle, which becomes monopolar.

### Tau excess induces poly and aneuploidy

**Fig. 3.**
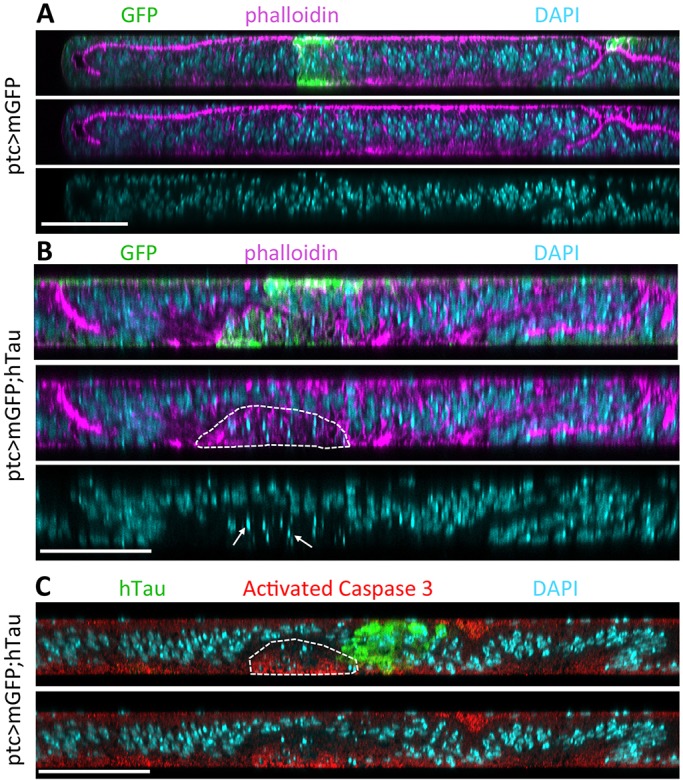
**hTau excess in the *ptc* area is associated with cell death at the basal side of the wing disc.** Immunostainings of dying epithelial cells in third instar larval wing discs overexpressing human Tau (hTau) protein 0N4R in the *ptc* domain. As a control, a membrane-targeted GFP (mGFP) is expressed alone in the same region. (A,B) The wing disc is shown in transverse confocal sections and the *ptc* area is visible in control (A) and hTau-overexpressing (B) conditions because of the presence of mGFP. (B) Triple staining (GFP: green, phalloidin: magenta, and DAPI: cyan) shows the presence of small spots of DAPI staining at the basal side of the wing disc within the GFP-positive area in the presence of hTau (arrows and encircled area). These small spots are indicative of dying cells. (C) The presence of dying cells at the basal side of the disc was further tested using activated-caspase-3 staining in discs overexpressing hTau with *ptc-Gal4*. Transverse confocal sections of a wing disc stained for hTau (green), activated caspase 3 (red) and DAPI (cyan) shows the presence of activated-caspase-3 staining in the area where cells are dying, as indicated by DAPI bright spots (encircled area) at the basal side of the wing disc. Scale bars: 50 µm. These experiments were replicated at least three times in the laboratory.

**Fig. 4.**
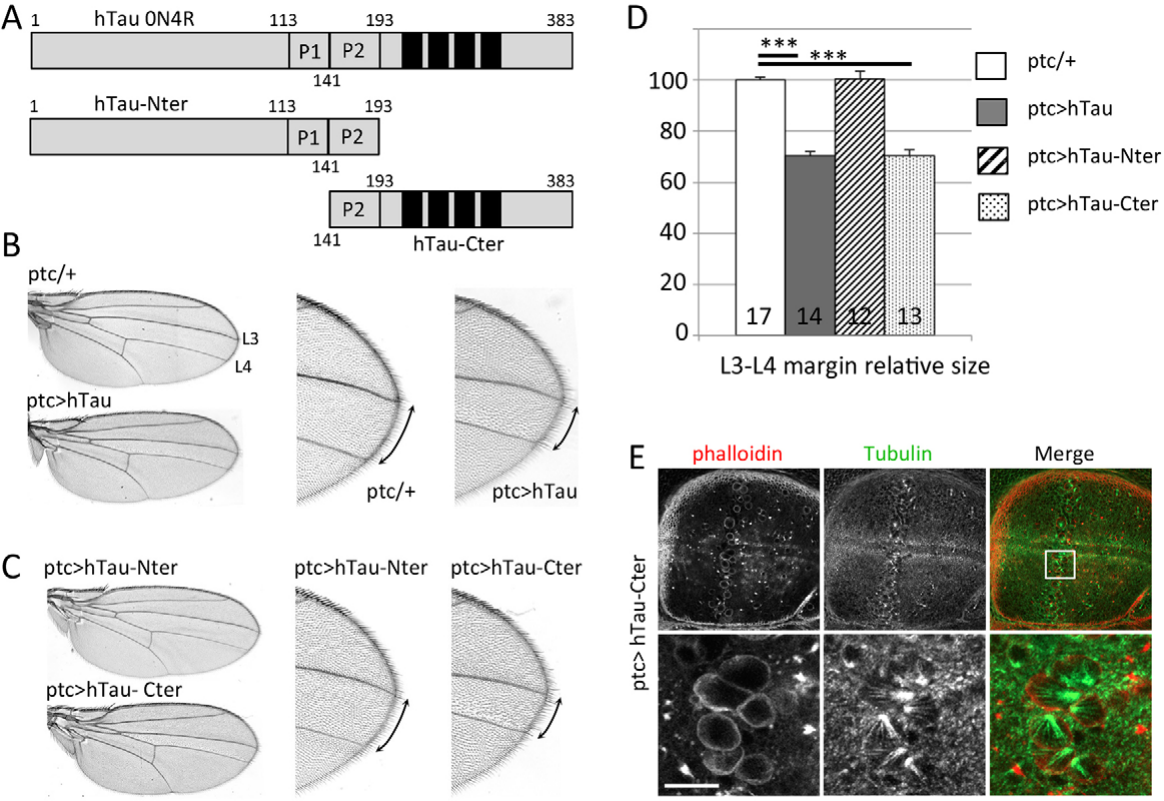
**Adult wing phenotypes induced by full-length or truncated hTau expression with *ptc-Gal4* and their correlation with the presence of monopolar spindles at the larval stage.** (A) Description of the different 0N4R hTau constructs used. (B) Representative adult wing images from control (*ptc/+*) and flies overexpressing full-length 0N4R hTau (*ptc>hTau*): the L3-L4 intervein, where lies the domain of *ptc-Gal4* expression, is smaller in presence of hTau. Higher magnification of the wing margin highlights this difference and also shows mis-oriented cell hairs near L4. (C) Representative adult wing images from flies overexpressing hTau-Nter (*ptc>hTau-Nter*) or hTau-Cter (*ptc>hTau-Cter*): the L3-L4 intervein size is unchanged in the presence of hTau-Nter, but is reduced in the presence of hTau-Cter. Higher magnification of the wing margin highlights the phenotypes and also shows mis-oriented cell hairs near L4 in the presence of hTau-Cter, similarly to what is seen with full-length hTau. (D) Quantification of the L3-L4 intervein size (margin), showing that overexpression of hTau-Cter gives the same significant phenotype as overexpression of full-length hTau (data expressed in % of control genotype). Histogram shows mean values±s.e.m. for the set of measurements. The number of wings measured for each genotype is indicated at the bottom of each histogram bar. Two-tailed Student's *t*-test was performed to compare mutant genotypes with the control genotype. ***P<0.001. (E) Representative immunostaining of mitotic spindles in third instar larval wing discs overexpressing hTau-Cter in the *ptc* domain: Tubulin (green) and phalloidin (red). Similarly to what is observed in the presence of full-length hTau, the expression of hTau-Cter induces monopolar spindles in the *ptc* domain of expression. Scale bar for the top panels of E is 50 µm and 10 µm for the bottom panels. These experiments analyzing adult wing phenotypes were replicated at least three times, giving the same phenotypes. Corresponding measurements were made twice.

We then tested whether this had consequences on chromosome segregation. We looked at the chromosomes by staining them with PH3 and could see that some cells had an increased and abnormal number of chromosomes (Fig. 2C). Indeed, because imaginal disc cells are diploid (Fuse et al., 1994), the maximum number of chromosomes should be eight (four chromosomes segregating in each daughter cell). In the presence of hTau excess, several cells contained largely more than eight chromosomes. One example is shown in detail in Fig. 2C, in which chromosomes were counted one by one (each new chromosome that is visible on the next focal plane is labelled with a star). For the selected cell, the total number of chromosomes was 20. Knowing that there is no particular change in S phase, as assessed by BrdU staining, this indicates that some cells are mitotically blocked, but have undergone new S phase. The fact that the number of chromosomes is variable and is not a factor of four could be explained by the presence of some cytokinesis occurring in cells with an abnormal spindle, which would lead to daughter cells with an abnormal number of chromosomes. It is known that aneuploidy often leads to apoptotic cell death (Peterson et al., 2012). Also, it has been shown that defective alignment of the mitotic spindle in the wing disc correlates with cell delamination and apoptotic death at the basal face of the disc (Nakajima et al., 2013). Hence, if cytokinesis occurs in cells with a misaligned monopolar spindle, the most basal daughter cell will probably undergo apoptotic cell death. To see whether such cell death occurred in the hTau-overexpressing domain, we looked at transverse *z*-sections of the wing disc. We could indeed see apoptotic fragments of nuclei, as stained with DAPI, specifically in the zone where an excess of Tau is present in the epithelial cells (Fig. 3). We also detected activated-caspase-3 staining in the hTau-overexpressing domain, at the basal surface of the epithelium, further confirming the presence of apoptotic cells delaminating from the epithelium (Fig. 3). We looked at adult wings in order to see whether such cell death occurring from the larval stage in the *ptc* domain could have an effect on the size of this domain in adult wings. This was indeed the case and the *ptc* domain (in intervein region between L3 and L4) was smaller in the presence of an excess of hTau (Fig. 4A-D).

In conclusion, our results show that an excess of hTau leads to spindle defects, abnormal chromosome segregation and apoptotic cell death.

### hTau C-terminal microtubule-binding domain is responsible for hTau-induced mitotic arrest

To get insight into the molecular mechanisms involved, and whether hTau binding to MTs was important for this effect, we tested which protein domain of hTau is responsible for this defect. Tau has different protein domains and can be subdivided into four regions: an N-terminal projection region, a proline-rich domain, an MT-binding domain (MBD) consisting of either three or four tandem repeat sequences (depending on alternative splicing) and a C-terminal region (Mandelkow et al., 1996). Tau's ability to bind MTs depends on the MBD as well as on adjacent regions (Gustke et al., 1994). More precisely, the repeat sequences within the MBD are thought to directly bind MTs through their positive net charge, which interacts with negatively charged residues in tubulin (Jho et al., 2010; Kar et al., 2003). Here, we constructed two partial sequences of hTau (Fig. 3A), one consisting of the N-terminal half of the protein, including the proline-rich domain (hTau-Nter1-193) and one consisting of the C-terminal half of the protein (hTau-Cter141-383), including part of the proline-rich domain, which was shown to be required for proper MT-binding of the MBD (Elie et al., 2015). Hence, only the C-terminal construct can bind to MTs. Transgenic lines were obtained with both constructs inserted at the same genomic position as was the full-length *hTau* transgene, in order to obtain a similar level of transgene expression. Also, all constructs, including wild-type *hTau*, are flag-tagged in the C-terminal, enabling determination of expression level (Fig. S4). We tested the effect of both constructs, by expressing them with the *ptc-Gal4* driver. When looking at adult wings, we could see that only the C-terminal domain induced a wing defect like that seen when overexpressing the full-length hTau (Fig. 4B-D). We further looked at larval wing discs overexpressing the C-terminal of hTau and could see the same monopolar spindle defects as those seen with full-length hTau (Fig. 4E). This suggests that hTau binding to MTs might be the cause of these spindle defects. We further verified this hypothesis by comparing the effect of two different full-length *hTau* transgenes, *hTau*^S2A^ and *hTau*^S11A^. The corresponding hTau proteins are mutated on different phosphorylation sites and are known to differ in their ability to bind MTs (Chatterjee et al., 2009): contrarily to hTau^S11A^, the hTau^S2A^ protein, which bears mutations within the MBD only, binds weakly to MTs. Hence, compared to hTau^S11A^, the expression of hTau^S2A^ should be less deleterious for mitosis if MT binding is required for the effect of hTau. When expressed in the whole wing discs (Fig. S5), we observed abnormal mitosis with monopolar spindles in the presence of hTau^S11A^, as we previously noticed with wild-type hTau. Interestingly, there was no obvious defect in mitosis in the presence of an excess of hTau^S2A^ (Fig. S5). This further confirms the importance of hTau binding to MTs as being the cause of the observed mitotic defects. hTau binding to MTs could affect mitosis in different ways: either hTau would overstabilize MTs and disrupt their normal dynamics during mitosis, or hTau would interfere with the function of other MT-binding proteins such as kinesins, which are important for normal cell division. In particular, hTau was shown to induce MT release from both kinesin-1 and Eg5 in gliding assays (Dixit et al., 2008; Ma et al., 2011). In addition, when testing the importance of more than 20 kinesin genes for cell division in *Drosophila* S2 cells (Goshima and Vale, 2003), it has been shown that loss of function of Klp10A, Ncd, Klp67A or Klp61F/Eg5 cause monopolar spindles, which is reminiscent of what we observed in wing discs overexpressing hTau.

### hTau-induced mitotic defects are similar to Klp61F (Eg5) loss-of-function defects

**Fig. 5.**
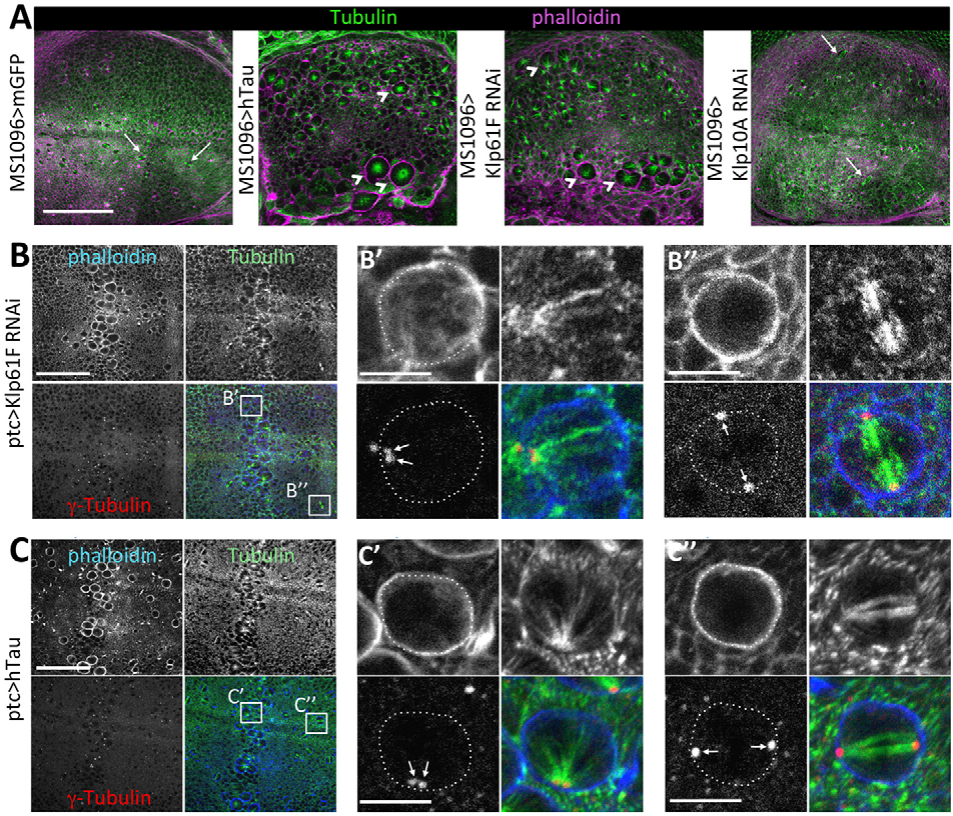
**Loss of function of the Klp61F kinesin in the wing disc induces monopolar spindles similarly to hTau overexpression, and hTau overexpression prevents separation of duplicated centrosomes as does the** **loss of Klp61F** **function.** (A) Immunostainings of third instar larval wing discs expressing membrane-targeted GFP (mGFP) or hTau or Klp61F-RNAi or Klp10A-RNAi in the whole wing pouch with the driver MS1096: Tubulin (green) and phalloidin (magenta). Left panel: control wing disc (with the expression of mGFP) shows no defects in cell division (some normal divisions are indicated by arrows). Middle left panel: when hTau is overexpressed with MS1096, many cells are blocked in mitosis with monopolar spindles (arrowheads). Middle right panel: when Klp61F expression is reduced in the whole wing disc by RNAi, there are many cells blocked in mitosis with monopolar spindles (arrowheads), a phenotype similar to the one obtained with hTau overexpression. Right panel: when Klp10A expression is reduced in the whole wing-disc by RNAi, there is no mitosis blocking with monopolar spindle and many normal cell divisions are present (see arrows). Scale bar: 100 µm. (B,C) Immunostainings of third instar larval wing discs expressing Klp61F-RNAi or hTau in the *ptc* domain of the wing disc: Tubulin (green), γ-Tubulin (red; to stain centrosomes) and phalloidin (blue). (B) In accordance with the known function of Klp61F, when its level of expression is reduced by RNAi in the *ptc* region, monopolar spindles are associated with duplicated centrosomes that do not move apart to each side of the cell (B′), whereas the centrosomes are associated with each spindle pole in the control area of the wing pouch (B″). Cell contours are outlined by a dotted line and centrosomes are indicated by arrows. (C) Overexpression of hTau in the *ptc* region gives the same phenotype as partial loss of function of Klp61F: duplicated centrosomes do not move apart in the presence of hTau (C′), whereas they move apart to each pole in the control area of the wing pouch (C″). Cell contours are outlined by a dotted line and centrosomes are indicated by arrows. Scale bars: 50 µm in B and C and 5 µm in B′, B″, C′ and C″. All these experiments were performed at least three times.

In order to investigate the hypothesis of hTau affecting kinesin function during mitosis, we looked at whether these specific kinesins are actually important for spindle dynamics in the wing disc. We tested the consequences of RNA interference (RNAi)-induced loss of function of these kinesins, using the *MS1096* driver (Fig. 5A and Fig. S6). Similarly to what is observed in the presence of hTau overexpression, we observed enlarged cells with monopolar spindles in wing discs expressing RNAi for *Klp61F*/*Eg5* (Fig. 5A). We did not detect such a phenotype in wing discs expressing RNAi for *Klp10A*, *Ncd* or Klp67A (Fig. 5A and Fig. S6). This result suggests that hTau excess might actually inhibit mitosis in the wing disc by inhibiting the function of the Klp61F/Eg5 kinesin. This Klp61F/Eg5 kinesin is a plus-end-directed tetrameric kinesin. Its antiparallel tetrameric organization is fundamentally different from the majority of other kinesins that are dimers, such as the well-characterized conventional kinesin-1. Because it is able to slide apart bundles of anti-parallel oriented MTs, this kinesin plays an important role during mitosis. In particular, it is important for the separation of duplicated centrosomes and their positioning at opposite poles of the dividing cells. In *klp61F* mutants, the centrosomes of dividing cells fail to migrate at each pole of the cell and are found adjacent to each other (Sharp et al., 1999). If hTau excess actually impairs mitosis by inhibiting Klp61F/Eg5 function, we should also see such defects of centrosome migration. We tested this by comparing centrosome position in wing discs expressing either hTau in excess or a *Klp61F* RNAi construct in the *ptc* area. Both conditions gave the same phenotype: cells within the *ptc* area displayed monopolar spindles with duplicated and unseparated centrosomes (Fig. 5B-C″). This result further suggests that hTau blocking of mitosis might occur via Klp61F inhibition. We then tested whether we could find this negative interaction between hTau and Klp61F by genetic means.

### Genetic interaction between Klp61F/Eg5 and hTau

**Fig. 6.**
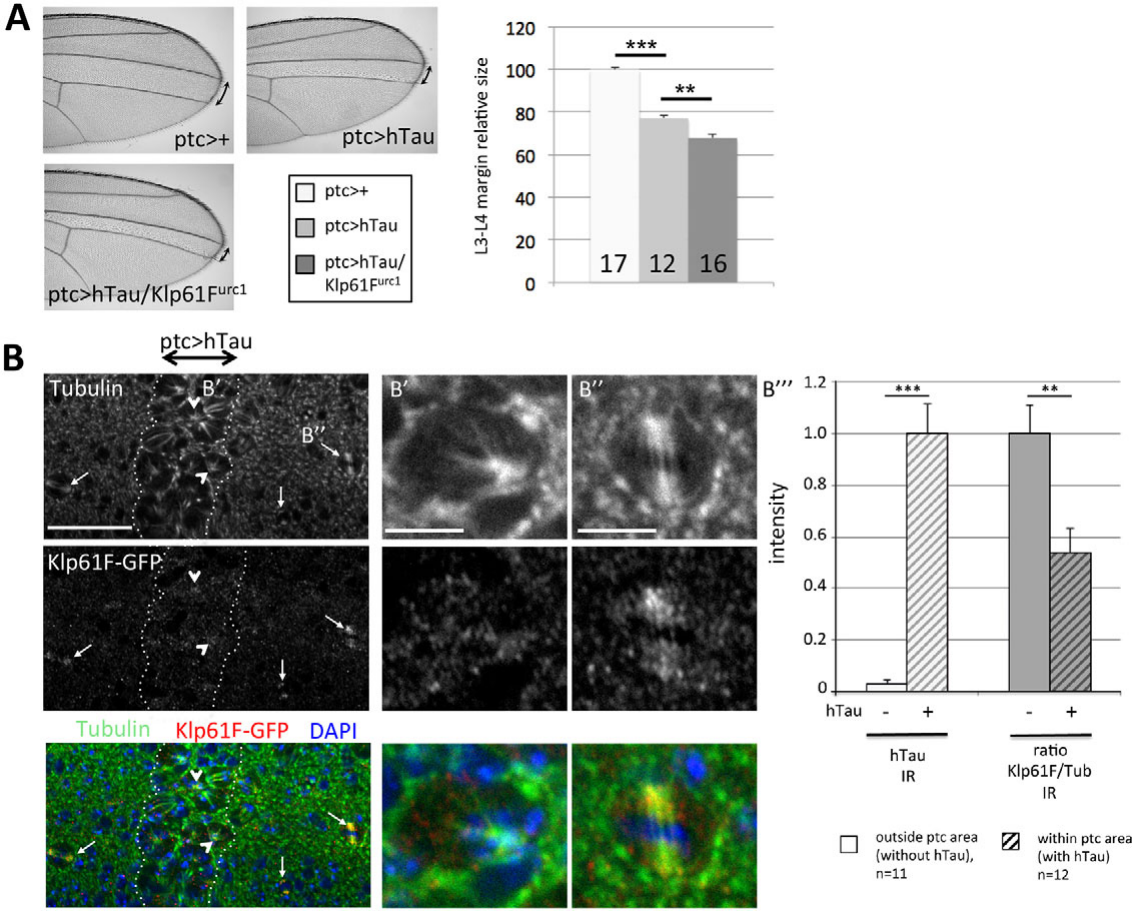
**Genetic interaction between hTau overexpression and *Klp61F* in the wing, and loss of Klp61F colocalization with microtubules in the presence of hTau.** (A) Representative adult wing of control flies (*ptc-Gal4/+*), or flies overexpressing hTau (*ptc-Gal4/+;UAS-hTau/+*), or flies overexpressing hTau in the presence of a transheterozygous loss-of-function mutation of *Klp61F* (*ptc-Gal4/+; UAS-hTau/Klp61F^urc1^*). Quantification of the length of the L3-L4 intervein margin in these different genotypes shows that the hTau-induced defect is significantly enhanced by half-dose reduction of the *Klp61F* gene. ***P<0.001, **P<0.01. The number of wings analyzed for each genotype is indicated at the bottom of each histogram bar. (B) Representative immunostaining of third instar larval wing discs expressing Klp61F-GFP ubiquitously in the wing disc and hTau specifically in the *ptc* area (within dotted lines) (*ptc-Gal4/+;UAS-hTau/ubi-Klp61F-GFP*): Tubulin (green), Klp61F-GFP (red) and DAPI (blue). Arrows indicate control dividing cells (outside the *ptc* area) in which there is high Klp61F-GFP immunoreactivity colocalizing with spindle microtubules. Arrowheads indicate monopolar spindles of dividing cells in the presence of an excess of hTau (within the *ptc* area): there is a low level of Klp61F-GFP immunoreactivity colocalizing with spindle microtubules in these cells. Scale bar: 30 µm. A higher magnification of such a cell with a monopolar spindle is shown in B′. A higher magnification of a control cell, which divides normally with a high amount of Klp61F-GFP colocalizing with the mitotic spindle, is shown in B″. Scale bars: 5 µm. The quantification of the relative intensity of Klp61F-GFP staining along microtubules in dividing cells within the *ptc* area or outside the *ptc* area in *ptc-Gal4/+;UAS-hTau/ubi-Klp61F-GFP* wing discs is shown in B‴: there is significantly less Klp61F immunoreactivity (IR) colocalizing with microtubules in the presence of hTau. As a control for hTau expression, these discs were immunostained for hTau in parallel to Klp61F-GFP and Tubulin, and the level of hTau immunoreactivity quantified for each cell measured within the *ptc* area (*n*=12 cells) or outside the *ptc* area (*n*=11 cells). Data are means±s.e.m. of intensity measurements on different wing discs (*n* indicated in the graph). Two-tailed Student's *t*-test was performed to compare the two conditions. **P<0.01, ***P<0.001. All staining experiments were performed at least three times.

We tested for a negative interaction between hTau and Klp61F, looking at adult wings obtained from individuals overexpressing hTau within the *ptc* area. In these conditions, the L3-L4 intervein area is reduced because of the important cell death that occurs consecutively to the mitotic blocking induced by hTau. We tested whether loss of one copy of the *Klp61F* gene could accentuate the hTau-induced reduction in intervein size. This was indeed the case, as shown in Fig. 6A. This genetic interaction further suggests that hTau-induced defects are the consequences of Klp61F/Eg5 inhibition and raises the question of the molecular mechanisms involved. Because previous work showed that hTau could induce MT release from Eg5 in gliding assays (Ma et al., 2011), we tested whether the hTau effect in the wing disc could actually be the consequence of Klp61F/Eg5 detachment from MTs.

### Klp61F/Eg5 localization is modified in the presence of an excess of hTau

The mechanism of interaction between Klp61F/Eg5 and hTau might be similar to the one described by Dixit et al. for kinesin-1 and hTau: kinesin-1 movement along MTs is stopped when encountering Tau protein bound to MTs, and this leads to the detachment of kinesin-1 from MTs (Dixit et al., 2008). If the situation were similar for Klp61F/Eg5, we should see less Klp61F/Eg5 bound to MTs during mitosis in the presence of hTau. We tested this by comparing Klp61F localization in dividing cells overexpressing hTau or not within the wing disc. Outside the *ptc* area, control dividing cells are found with a high content of Klp61F colocalizing with mitotic MTs (Fig. 6B and Fig. S7). This is not the case within the *ptc* area, where hTau is expressed and where dividing cells have a much lower amount of Klp61F colocalizing with mitotic MTs (Fig. 6B and Fig. S7). This result not only confirms a functional interaction between hTau and Klp61F/Eg5, but also provides *in vivo* evidence that hTau excess actually affects Klp61F localization to mitotic MTs. Hence, mitosis blocking in the presence of hTau excess would be the consequence of the inhibition of Klp61F/Eg5 movement along MTs and its detachment from MTs.

### hTau excess also affects mitosis in neuronal tissues

**Fig. 7.**
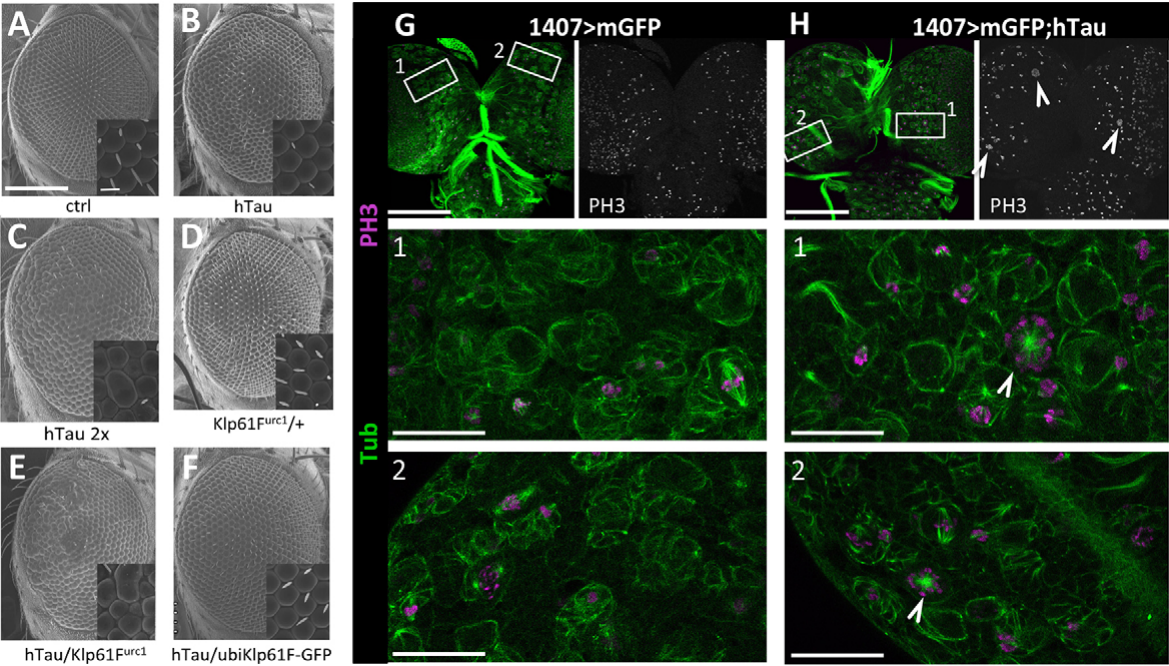
**Effects of hTau overexpression in tissues from the nervous system: eye roughness and genetic interaction with *Klp61F* in the eye, monopolar spindles in larval neuroblasts.** (A-F) Eye roughness induced by an excess of hTau driven by *GMR-Gal4*. Representative adult eye, as viewed by scanning electron microscopy, of control flies (*GMR-Gal4/+*) (A), or flies overexpressing one dose of hTau (*GMR-Gal4/+;UAS-hTau/+*) (B), or flies overexpressing two doses of hTau (*GMR-Gal4/+;UAS-hTau/UAS-hTau*) (C), or flies transheterozygous for the mutation *Klp61F^urc1^* (*Klp61F^urc1^/+*) (D), or flies overexpressing one dose of hTau in a transheterozygous background for *Klp61F^urc1^* (*GMR-Gal4/+; UAS-hTau/Klp61F^urc1^*) (E), or flies overexpressing one dose of hTau in the presence of ubiquitous overexpression of Klp61F-GFP (*GMR-Gal4/+; UAS-hTau/ubi-Klp61F-GFP*) (F). Both control genotypes (A and D) show normal eyes. The expression of hTau in the developing eye induces loss of adult eye bristles (one dose of hTau, in B) and ommatidial fusion (two doses of hTau, in C). Half-dose for *Klp61F* increases the eye phenotype induced by hTau (with the appearance of ommatidial fusion in the presence of one dose of hTau, E). Increasing the amount of Klp61F, by its overexpression using the ubiquitin promotor, leads to a phenotypic rescue of the hTau-induced loss of bristles (F). Scale bar: 100 µm and 10 µm (inserts). These experiments were replicated at least three times. (G,H) Monopolar spindles induced by hTau overexpression in larval neuroblasts. Immunostainings of mitotic spindles and mitotic chromosomes in third instar larval brains overexpressing either mGFP alone or mGFP and hTau in neuroblasts with the *Gal4* driver 1407: Tubulin (green), PH3 (magenta). (G) The PH3 pattern in control brains (mGFP expression) does not show any neuroblast with an abnormal number of chromosomes. High magnification of neuroblasts from regions 1 and 2 shows that most mitotic spindles of dividing neuroblasts are parallel to the brain surface. (H) The PH3 pattern in brains overexpressing hTau in neuroblasts shows cells with an abnormal number of PH3-positive chromosomes (see arrowheads). High magnification of these cells from regions 1 and 2 shows that these are neuroblasts with monopolar spindles (arrowheads), with the same aspect as in the wing disc when hTau is overexpressed. Scale bars: top panels is 100 µm and 20 µm in panels 1 and 2. These experiments were replicated at least three times.

The question is now open to see whether such an effect of hTau excess in mitosis can be found in other tissues than the wing disc epithelium. To answer this question, and to focus on neuronal tissues, we first looked at whether hTau excess induced mitotic defects in another imaginal disc, the eye disc. This tissue gives rise to photoreceptors, which are neuronal cells. The cell division pattern within the eye disc is more complex than within the wing disc, with the presence of a morphogenetic furrow associated with two mitotic waves. It is known that inhibition of cell division within the eye disc (i.e. the second mitotic wave using the *GMR* promoter) leads to loss of bristles, fusion of ommatidias and eye roughness (Morris et al., 2001). Here, we tested whether the known eye roughness induced by overexpression of hTau in the developing eye disc with the *GMR-Gal4* driver (Jackson et al., 2002; Wittmann et al., 2001) is due to Klp61F-related cell cycle defects. We tested whether loss of one copy of the *klp61F* gene increased the eye phenotype. The expression of one dose of our *hTau* transgene gave a mild eye phenotype with only bristles missing, and almost no ommatidial disorganization (Fig. 7A,B), whereas the expression of two copies of the *hTau* transgene led to a stronger phenotype, with more bristles missing and ommatidial disorganization at the posterior side of the eye (Fig. 7C). Loss of one copy of *klp61F* in the presence of one dose of hTau leads to a phenotype that is similar to, and even stronger than, the one observed with two copies of the *hTau* transgene (Fig. 7D,E). This confirms the genetic interaction observed when expressing hTau in the wing disc. We further tested whether overexpression of Klp61F-GFP under the ubiquitin promoter could rescue the bristle phenotype observed in the presence of one copy of the *hTau* transgene. This was indeed the case, with almost no bristles missing in this genetic condition (Fig. 7F). Also, the MBD of hTau, but not the N-terminal projection domain, induced the same eye phenotype as full-length hTau (Fig. S8), as was expected from their effect on mitosis in the wing disc. Altogether, these results provide evidence that the effect of hTau on cell division, through Klp61F dysfunction, can also be found in the eye disc.

To further study the effect of hTau on mitosis in neuronal tissues, we focused on dividing neuroblasts in the larval brain. Note that it was previously shown that *klp61F* mutants presented mitotic defects in neuroblasts, with monopolar spindles and clear aneuploidy (Heck et al., 1993). We used the pan-neuroblast 1407 *Gal4* driver (Luo et al., 1994) to overexpress hTau in neuroblasts and see whether this led to mitosis arrest with monopolar spindles and chromosome aneuploidy. We could indeed see these defects when comparing control brains (Fig. 7G) with brains overexpressing hTau (Fig. 7H): abnormal mitosis with monopolar spindles and chromosomal aberrations could only be seen in the presence of hTau. This indicates that the toxic effect of hTau on mitosis is not specific to epithelial cells from imaginal discs but can also be found in dividing precursors of the central nervous system.

### The interaction between Tau and Klp61F/Eg5 is conserved between *Drosophila* and humans

**Fig. 8.**
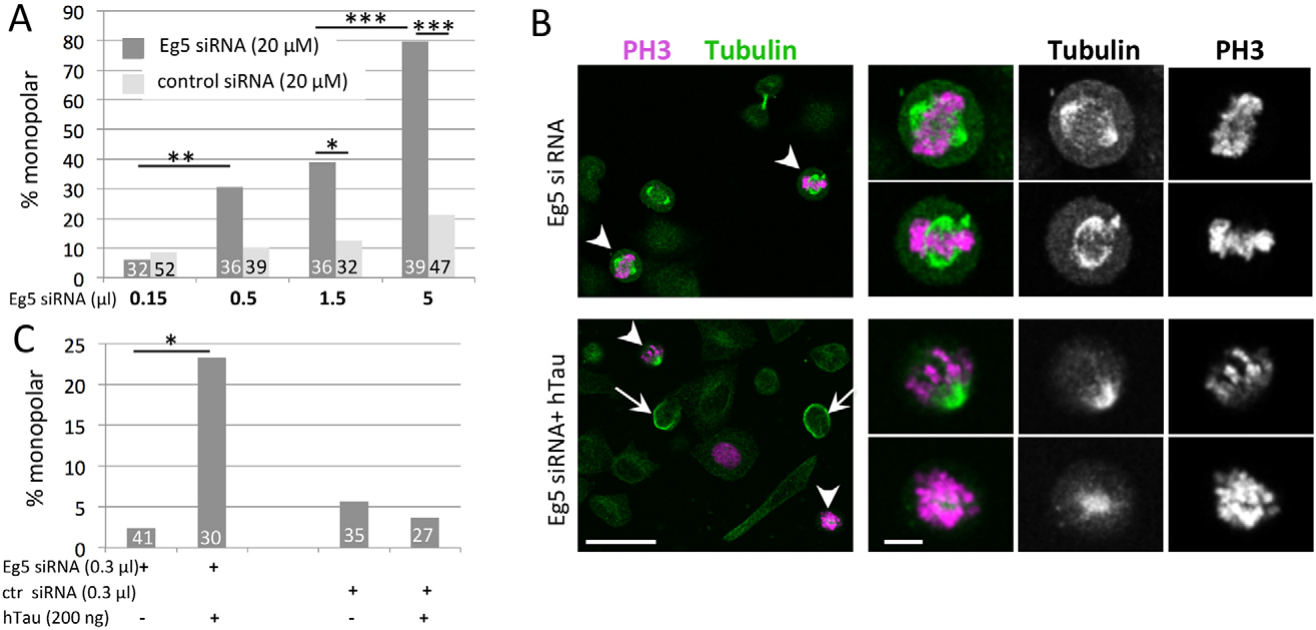
**hTau expression enhances the monopolar spindle phenotype induced by *Eg5* knockdown in HeLa cells.** (A) Quantification of the percentage of PH3-positive cells having a monopolar spindle when transfecting increasing amounts of *Eg5*-siRNA or control siRNA (mutated sequence). (B) Representative immunostaining of HeLa cells transfected with low amounts of *Eg5* siRNA (0.3 µl of a 20 µM solution, i.e. 80 ng) only (top panels) or with 200 ng of *hTau* DNA in addition (bottom panels): Tubulin (green), PH3 (magenta). PH3-positive cells are indicated by arrowheads and are shown in the higher-magnification panels. In conditions of transfection with a low amount of *Eg5* siRNA, more than 90% of cell divisions display normal bipolar spindles, and abnormal cell divisions with a monopolar spindle are rarely found. When 200 ng *hTau* is co-transfected, we can detect bundles of microtubules in some cells (arrows). In addition, among PH3-positive dividing cells, 20% of the cells display abnormal monopolar spindles (arrowheads), indicating a positive interaction between loss of Eg5 function and hTau overexpression. (C) Quantification of the percentage of PH3-positive cells with monopolar spindles in the different conditions of transfection of one representative experiment. Number of cells counted for each condition is noted at the bottom of each histogram bar. Statistical tests were chi-2 tests. *P<0.5, **P<0.01 and ***P<0.001. Note that co-transfection of *hTau* in the presence of low amount of *Eg5* siRNA significantly increases the percentage of cells with a monopolar spindle, whereas it has no effect in the presence of the same amount of control siRNA. This indicates that, at this concentration, the expression of hTau alone has no effect on the percentage of monopolar spindles, whereas it gives an effect when the amount of Eg5 is concomitantly knocked-down in the cells. Scale bars: 60 µm (left panels) and 10 µm (right panels). These experiments were replicated three times.

Altogether, our results indicate that hTau can inhibit Klp61F function in *Drosophila* cells. Although it was previously shown that hTau could actually inhibit Eg5 function *in vitro* (Ma et al., 2011), we further wanted to test whether this was indeed the case in human cells. Hence, we tested whether the expression of hTau in HeLa cells could actually enhance the monopolar spindle phenotype previously reported when Eg5 expression was inhibited with siRNAs (Zhu et al., 2005). Using siRNAs targeting *Eg5* at increasing doses, we observed an increasing percentage of monopolar spindles among mitotic (PH3-positive) cells (Fig. 8A). There was no significant increase of mitotic cells with monopolar spindles when using control mutated siRNAs (Fig. 8A). When additionally transfecting *hTau* onto cells treated with the lowest doses of siRNA, we found an increase in the percentage of monopolar spindles, which was not the case in conditions using control siRNAs (Fig. 8B,C). This indicates that the presence of an excess of hTau actually enhances the defects induced by Eg5 knockdown, confirming that the interaction between hTau and Klp61F/Eg5 observed either *in vitro* or in *Drosophila* can also be detected in human cell lines.

Reciprocally, we tested whether the mitotic blockade induced by hTau in *Drosophila* dividing cells could also be induced by an excess of *Drosophila* Tau. When we overexpressed *Drosophila* Tau in the wing disc in the same conditions as hTau, we could also detect mitotic defects with monopolar spindles as well as a reduction of the wing size (Fig. S9). In addition, we found the same genetic interaction with *Klp61F* as seen between *Klp61F* and hTau (Fig. S9). This indicates that the interaction between Tau and Klp61F/Eg5 is conserved among species.

## DISCUSSION

Here, we report for the first time that an excess of hTau in dividing cells leads to a mitotic arrest of these cells, associated with the presence of monopolar spindles, aneuploidy and cell death. hTau protein has two paralogous proteins in vertebrates, namely MAP2 (preferentially localized in the somatodendritic compartment of neurons) and MAP4, which is rather ubiquitously expressed (Morris et al., 2011). All of them are highly conserved in the sequence of their MBD (Dehmelt and Halpain, 2005). A negative effect of MAP4 on mitosis was previously reported (Holmfeldt et al., 2003): when transiently induced in human K562 erythroleukemia cells, DG75 Burkitt's lymphoma cells or Jurkat acute T-cells leukemia, MAP4 induced mitotic arrest with the presence of monopolar spindles. This conserved effect between MAP4 and hTau in blocking mitosis is in accordance with the implication of the MT-binding repeats, which are the most conserved sequences between the two proteins. It is surprising that this effect on mitosis was never reported for the protein hTau when transfected in cells in culture, considering the number of publications and teams having performed these experiments (Frost et al., 2015; Morris et al., 2011). One reason could be that very few studies actually focused on mitosis and the mitotic spindle in the presence of an excess of hTau. A recent study focused on the effect of an excess of Tau on S phase: there was no significant effect for all hTau isoforms tested, except a 10% reduction in S phase observed with an excess of the 1N3R isoform (Li et al., 2015). In our hands, using HeLa cells, we used low concentrations of hTau plasmid and could not detect any change in the amount of cell divisions with monopolar spindles at these concentrations. In addition, there is a negative correlation between the presence of hTau and the number of dividing cells visible within the well. The causes might be that hTau-expressing cells die before dividing or that hTau-expressing cells stay in the G0 or quiescent phase and do not enter the cell cycle. Thus, specific transient expression conditions would be required to observe hTau-induced mitotic blocking in culture before cell death or quiescence occurs.

Our study is, to our knowledge, the first one describing transgenic flies with transgenes expressing fragments of hTau, all inserted at the same genomic position using the PhiC31 integrase system (Bischof et al., 2007), with the aim to reduce the differential positional effects for each transgene and to be able to compare the phenotypes obtained between each transgene. Getting rid of positional effects was indeed shown to be particularly important when comparing the severity of phenotypes for full-length hTau overexpression (Povellato et al., 2014). We were thus able to show that the expression of the C-terminal half of hTau (141-383) was able, on its own, to induce the same mitotic blocking and the same adult wing or eye phenotype than the expression of full-length 0N4R hTau (1-383), and that the expression of the N-terminal half of 0N4R hTau (1-193) had no effect on mitosis and no wing or eye phenotype. This led us to conclude that hTau effect might be due to its binding to MTs. These results are in accordance with the recently published paper from Geng et al., in which *hTau* transgenes were randomly inserted within the genome and the level of protein expression estimated by western blot (Geng et al., 2015). This study showed that *GMR*-driven expression of the C-terminal part of 2N4R hTau (231-441), which corresponds to the region 127-383 of 0N4R hTau, induced the same rough-eye phenotype as the *GMR*-induced expression of full-length 2N4R hTau protein. Altogether, this confirms the importance of the C-terminal moiety of hTau in the cellular toxicity of this protein.

Our description of the deleterious role of hTau on the mitotic spindle makes sense when considering the previously published results indicating that hTau is phosphorylated during mitosis (Preuss et al., 1995; Vincent et al., 1996). Remarkably, hTau is phosphorylated, during mitosis, such as to give the same immunoreactive pattern as hyperphosphorylated ‘pathological’ Tau in AD, using the antibodies anti-AT8, -Tau-1, -T46 and -PHF-1 (Preuss et al., 1995). Some of the phosphorylated residues are T153, T181, S202/205, T212/217 and S214, the latter having been shown to be important for MT binding of Tau (Illenberger et al., 1998). This hTau phosphorylation is associated to a change in protein localization: during mitosis, a substantial fraction of hTau is not bound to MTs, but retained in the cytoplasm. These results are in accordance with the idea that hTau binding to MTs is deleterious for mitosis, and that normal cellular physiology phosphorylates Tau during mitosis such as to avoid the mitotic blocking that we describe here. Whether mutating these phosphorylation sites would increase hTau-induced mitotic defects is an open question. Indeed, many mutations of Ser or Thr residues in Ala (either alone, or in combination) of hTau were tested for their deleterious effect when expressed in the *Drosophila* eye (Chatterjee et al., 2009; Fulga et al., 2007; Povellato et al., 2014; Steinhilb et al., 2007a,b) or in other tissues (Ubhi et al., 2007). The obtained results suggested that many phosphorylation sites within hTau could compensate for the mutation of one or several sites normally phosphorylated during mitosis. This functional redundancy demonstrates how physiologically important Tau phosphorylation is for normal cell biology. Note that S262 and S356, which are mutated in hTau^S2A^, are not residues strongly phosphorylated during mitosis (Illenberger et al., 1998). Hence, apart from being useful to correlate mitotic defects with hTau binding to MTs, this mutated construct has no relevance to the matter of endogenous phosphorylation of hTau during mitosis.

Our results show the presence of aneuploidy/hyperploidy in cells overexpressing hTau. This and other results are in favour of the chromosome missegregation/MT dysfunction hypothesis of AD, with the idea that, over a lifetime, defective mitoses lead to the accumulation of aneuploid cells throughout the body, including the brain, and that these aneuploid cells are more prone to neurodegeneration (Potter, 1991). These defective mitoses could occur during embryonic neurogenesis, or during adult neurogenesis. It is known that there is some constitutional polyploidy derived from chromosome missegregation during mitosis in neuronal progenitor cells (Iourov et al., 2009; Mosch et al., 2007): one study showed that about 10% of neurons from the entorhinal cortex have a DNA content between 2n and 4n in normal adult brains, while being negative for cyclin B1 staining (i.e. they were not undergoing an S-G2 transition phase) (Mosch et al., 2007). Several studies reported increased aneuploidy in the brain as well as in peripheral cells of individuals with AD (Iourov et al., 2009; Migliore et al., 1997; Mosch et al., 2007; Yurov et al., 2014). For example, Mosch et al. (2007) reported that individuals with AD had 20% of polyploid, cyclin-B1-negative neurons in the entorhinal cortex, raising the question of the mechanisms responsible for the increase in this population of neurons. Increased neuronal polyploidy was also observed in transgenic mice with mutated hTau (Rossi et al., 2014), as well as in *APP* and *PS1* mouse models (Boeras et al., 2008; Granic et al., 2010). There are two hypotheses explaining this increase: the first one being that neurons undergo new S phase during the course of the disease, especially during aging; the second one being that neurogenesis (embryonic, adult or both) is affected, leading to daughter cells with missegregation of chromosomes. Our data support the second hypothesis.

Interestingly, the amyloid Aβ1-42 peptide was also shown to disrupt the mitotic spindle (Borysov et al., 2011), although differently compared to the phenotypes we observed with hTau: incubation of *Xenopus* egg extracts with Aβ1-42 peptide induced shorter or bent mitotic spindles. This phenotype was rescued when adding recombinant motor domains of Eg5, KIF4A or MCAK. Similarly to what we show here, the effect of Aβ1-42 was shown to be due to its localization to the spindle and its interference with the normal association of Eg5, KIF4A and, to a lesser degree, MCAK. In our study, we focused only on kinesins, which generated a ‘monopolar spindle’ phenotype when knocked down in S2 cells (Klp10A/KIF2, Klp67A/KIF18, Klp61F/Eg5/KIF11 and Ncd/KIFC1) (Goshima and Vale, 2003). We found that Klp61F/Eg5 was the only one giving, when inhibited in the wing disc, a phenotype similar to hTau overexpression. Similarly, from these four kinesins, only Eg5/KIF11 and KIF2 gave monopolar spindles when its expression was inhibited with siRNA in HeLa cells (Zhu et al., 2005). Also, Klp61F was found as an enhancer of hTau toxicity from a genetic screen performed in the *Drosophila* eye: a P-element mutant (different from the mutant we used) of *Klp61F* enhanced the eye phenotype induced by overexpression of 2N4R hTau (Ambegaokar and Jackson, 2011). This independent result allows the generalization of our conclusions to the different isoforms of Tau, in accordance with the fact that mitosis blocking is observed with the C-terminal part of hTau only. In conclusion, our results, together with the previous biochemical demonstration that hTau affects Eg5 function on MTs (Dixit et al., 2008; Ma et al., 2011), indicate that the hTau defect *in vivo* is, at least partly, due to interference with Klp61F/Eg5 normal function. Hence, Aβ- and Tau-induced defects both converge on Eg5 dysfunction. The importance of Eg5 dysfunction might be related to the fact that, from the different kinesins we considered, only Eg5/KIF11 lies in a genetic region associated with increased risk for AD (Ertekin-Taner et al., 2004; Feuk et al., 2005; Prince et al., 2003; Reitz et al., 2012). Altogether, this suggests that Eg5/KIF11 dysfunction might play a role in AD and that protecting or increasing the activity of this kinesin could be one strategy to consider in order to modify the progression of the disease.

Because AD is not generally considered as a developmental disease, i.e. as a disease appearing in the aged individual as a consequence of developmental defects occurring during the division of neuronal precursors, but as a disease resulting from the aging and degeneration of post-mitotic neurons, it is important to also consider the Tau-Eg5 interaction in post-mitotic neurons. The study of Eg5 expression and localization in the brain did show that Eg5 was highly expressed during embryonic development, but was also found in the adult brain (Ferhat et al., 1998; Lin et al., 2011). In particular, it was found at higher levels in hippocampal neurons compared to sympathetic neurons (Ferhat et al., 1998). Two pieces of evidence suggest that Eg5 plays a role in intracellular transport or synaptic plasticity in post-mitotic neurons. The first one is that an excess of β-amyloid peptide reduces transport of neurotrophin and neurotransmitter receptors to the cell surface, via the inhibition of Eg5 function (Ari et al., 2014). In addition, inhibition of Eg5 with monastrol affects long-term potentiation (Ari et al., 2014). The second one is that Eg5 directly interacts with ZBP1, a protein involved in the transport of messenger ribonucleoproteins (mRNPs), resulting in abnormal transport of β-actin mRNA (Song et al., 2015). Knowing the importance of the actin cytoskeleton in synaptic plasticity processes (Bellot et al., 2014), this is another indication of a potential role of Eg5 in adult and aging post-mitotic neurons, which might be affected by the excess of Tau observed in individuals with AD.

In conclusion, our work, by demonstrating that MT-bound Tau inhibits kinesin-5 and cell mitosis, provides a new framework to consider the role of an excess of Tau either during neurogenesis or in unknown Eg5-dependent processes in post-mitotic neurons.

## MATERIALS AND METHODS

### Fly stocks

We used the following *Gal4* activator strains: *ptc*-*Gal4*, *dpp*-*Gal4*, *MS1096*-*Gal4*, *GMR*-*Gal4* (gifts from Sophie Layalle, IGF, Montpellier, France) and 1407-Gal4 *inscuteable* (*insc*)-gal4 (FBst0008751).

For the visualization of expression domains, we used a UAS-mCD8-GFP strain (FBst0005137) and, for the visualization of the localization of Klp61F protein, the Ubi-Klp61F.GFP strains (FBst0035509 and FBst0035510). To test genetic interactions with *Klp61F*, we used the loss-of-function mutant *Klp61F^urc-1^* (FBst0035508). The *UAS-hTau*^S2A^ (FBst0051365) and *UAS-hTau*^S11A^ (FBst0051366) strains were used to confirm the importance of Tau binding to MTs in the studied phenotypes. All these lines were obtained from the Bloomington *Drosophila* Stock Center.

For RNAi experiments, we used lines obtained from the Vienna *Drosophila* RNAi Center (Dietzl et al., 2007) containing an RNAi construct targeting the following genes: *Klp61F* (v109280, v52549), *ncd* (v110355, v22570), *Klp10A* (v41534) or *Klp67A* (v108852, v52105).

In order to overexpress *Drosophila* Tau, the UAS-dtau strain was a gift from E. Skoulakis (Mershin et al., 2004).

Third instar stage larvae were used independently of their sex, except for crosses with the *MS1096* driver (on the X chromosome), for which we selected males. Adults aged between 2 and 10 days old were selected for analysis of wing size and eye phenotypes. The phenotypes were similar in males and females. Quantifications of wing size were made on females. Eye pictures were obtained from females.

### Cloning and generation of transgenic fly lines

We obtained the pENTR-Tau vector containing the human 0N4RTau full coding sequence (1152 nt) from the Orfeome. Sequences corresponding to hTau N-terminal part (420 nt) or hTau C-terminal part (732 nt) were amplified by PCR and cloned in the Gateway pDONR221 vector by BP reaction (Gateway Technology) giving rise to the pENTR-hTau-CTer and pENTR-hTau-NTer vectors. The different ENTRY constructs were subcloned into the Gateway pUAST-WF-attB destination vector by LR reaction (Gateway Technology). The pUAST-WF-attB vector was derived from the Gateway vectors: pAWF (Invitrogen) and pUAST-W-attB (gift from Amira Brighi, IJM, Paris, France) in order to get a vector with attB recombination sites for site-specific integration in *Drosophila* and the Flag tag in the C-terminus of the subcloned protein fragments. Transgenic flies were generated with the site-specific phiC31 integration system (Bestgene Inc.) using ZH-attP-68E1 (24485 line) embryos (Bischof et al., 2007).

### Immunohistochemistry and BrdU labelling

#### Immunohistochemistry

Third instar larval imaginal discs and brains were dissected in PBS 1× and fixed for 20 min in 4% paraformaldehyde. After a wash in PBS 1×, Triton 0.3% (PBS-T), discs and brains were incubated for 1 h at room temperature with primary antibodies diluted in PBS-T, 0.3% BSA. Fluorescent secondary antibodies were used at the recommended dilution and incubated for 1 h. Preparations were mounted in ProlonGold media for observation.

#### BrdU labelling of discs

Third instar larval imaginal wing discs were grown in Schneider's medium (Invitrogen). BrdU was added to a final concentration of 100 μg/ml. After 1-3 h incubation, discs were washed with PBS-T and fixed for 20 min in 4% PFA. After PBS-T wash, discs were dissected and incubated in sodium citrate 10 mM (pH 6): PBS-T for 20 min at 95-100°C. Discs were allowed to cool for 20 min and blocked with PBS-T, 0.3% BSA for 30 min before incubating discs with anti-BrdU (Developmental Studies Hybridoma Bank, 1:1000) for 1 h at room temperature. Next, the standard immunohistochemistry protocol was followed.

#### Antibodies

Primary antibodies were: rabbit polyclonal anti-Tau (Dako #A002401, 1:500), mouse monoclonal anti-FlagM2 (Sigma #F1804, 1:1000), mouse monoclonal anti-GFP (Roche #11814460001, 1:5000), sheep polyclonal anti-tubulin (ATN02, Cytoskeleton, 1:500), rat monoclonal anti-tubulin (CBL270, Millipore, 1:1000), rabbit anti-cleaved-caspase-3 (Cell Signaling #9661S, 1:1000), mouse anti-PH3 (phospho-Ser10, clone 3H10, 1:1000), rabbit anti-PH3 (phospho-Ser10+Thr11, ab32107, Abcam, 1:1000), rhodamine phalloidin (Invitrogen #R415, 1:3000) and rabbit polyclonal anti-dTau (1:1000) (kind gift from D. St Johnston) (Doerflinger et al., 2003).

Secondary antibodies were Alexa-Fluor-488, Alexa-Fluor-633 (Molecular Probes, Invitrogen), Cy3 and Cy5 (Jackson ImmunoResearch), all diluted 1:500.

### HeLa cell culture and transfection

siRNA transfection with oligofectamine (Invitrogen) was performed as recommended by the manufacturer. Briefly, 10^5^ cells per well were seeded in six-well plates 16 h before transfection. Transfection was performed using 3 µl of oligofectamine and the indicated amount of siRNA (ranging from 0.1 to 10 µl of 20 µM siRNA) in serum-free culture medium. Culture medium was changed 12 h after transfection and replaced with complete culture medium. *Eg5* and mutated *Eg5* siRNA sequences are from Weil et al. (2002), i.e. 5′-CUGAAGACCGUGAAGACAAUUU-3′ (*Eg5* siRNA) and 5′-CACCUCAUAUUCCUUAUCGUU-3′ (ctrl siRNA). siRNAs were purchased from Dharmacon.

DNA transfection with effectene (Qiagen) was performed as recommended by the manufacturer. Briefly, 10^5^ cells per well were seeded in six-well plates 16 h before transfection. Transfection was performed using 10 µl of effectene and the indicated amount of DNA construct (ranging from 100 to 400 ng) in serum-free culture medium. Culture medium was changed 12 h after transfection and replaced with complete culture medium. The *hTau* fragment was cloned in pdest47 plasmid by LR reaction (Gateway Technology). HeLa cells were obtained from Dr Yoan Arribat (INM, Montpellier) and were not tested for mycoplasma contamination.

For all transfection experiments, cells were fixed 24 h or 40 h post-transfection in 2% PFA for 10 min and stained as described above.

Quantifications were made by taking random, non-overlapping, images at 20× magnification, and by counting the number of cells with bipolar or abnormal (monopolar) spindles among the PH3-positive mitotic cells present within each image.

### Imaging

Confocal images were acquired using a Zeiss LSM780 confocal microscope (Montpellier RIO Imaging, Institute of Human Genetics) equipped with 488 nm, 561 nm and 633 nm lasers, and the corresponding dichroic and filter sets.

### Scanning electron microscopy

For scanning electron microscopy (SEM), whole adult flies were anesthetized with CO_2_ and then dehydrated through a graded ethanol series (25, 50, 75, 2×100%) with 24-h incubations at each step. The flies were then equilibrated with graded ethanol–hexamethyldisilazane, and then hexamethyldisilazane alone. Subsequently, the samples were sputter coated with an approximate 10-nm-thick gold film and then examined under a scanning electron microscope (Hitachi S4000, at CRIC and Montpellier Rio Imaging, Montpellier France) using a lens detector with an acceleration voltage of 20 kV at calibrated magnifications.

### Methodology and statistics

Measurement of PH3-positive pixels in *ptc-Gal4*/+*; UAS-mCD8GFP/UAS-hTau* larvae was made by thresholding the PH3 staining and then measuring the particle number and area within the *ptc* domain (based on GFP staining) and outside the *ptc* domain, with the ImageJ software. Total PH3-positive area was then divided by the size of the domain considered. Student's *t*-test was performed to compare the percentages between the two domains.

For the measurement of differences in wing size, sample size was calculated based on the measured standard deviation (4.7 for a mean of 100 for the control genotype) such as to be able to detect a 5% variation of wing size with a type I error of 5% and a power of 80%: 14 wings had to be measured. Measurements were made blind of the genotype and, after testing for normality, Student's *t*-test was performed to compare the control and mutant genotypes.

For the measurement of differences in the proportion of dividing cells with monopolar spindles, sample size was arbitrarily set up at minimum 30 dividing cells for the dose-response curves with increasing amounts of *Eg5* siRNA. With this size, it was possible to detect an increase of the percentage of monopolar cells ranging from 5 to 80%. We used the same sample size to perform the dose-response curve with increasing concentrations of control siRNA and to test for the effect of co-transfection with *hTau*. Images of cells were taken randomly within the slide, scanning the slide, looking at PH3-positive spots and then taking pictures of the tubulin staining in order to see the spindle. Statistical tests used are chi-2 tests.
